# New Bills of Mortality for London

**Published:** 1841-10

**Authors:** 


### PART FOURTH.

### Medical Intelligence.

### TABLE OF MORTALITY FOR LONDON,

### For the Second Quarter of 1841:

Showing the number of Deaths from all causes registered in twelve weeks, from the 27th March, 1841, to the 24th June, 1841.

| Causes of Death. | Total Deaths. |
|---|---|
| **Class I.** | |
| Smallpox | 247 |
| Measles | 142 |
| Scarlatina | 120 |
| Hooping-cough | 548 |
| Croup | 102 |
| Thrush | 54 |
| Diarrhœa | 68 |
| Dysentery | 12 |
| Cholera | 1 |
| Influenza | 78 |
| Typhus | 258 |
| Erysipelas | 55 |
| Syphilis | 7 |
| Hydrophobia | 1 |
| Total Epidemic, &c. | 1693 |
| **Class II.** | |
| Cephalitis | 173 |
| Hydrocephalus | 467 |
| Apoplexy | 214 |
| Paralysis | 158 |
| Convulsions | 650 |
| Epilepsy | 49 |
| Insanity | 9 |
| Delirium tremens | 20 |
| Dis. of brain, &c. | 128 |
| Total Dis. of Brain, &c. | 1868 |
| **Class III.** | |
| Quinsy | 20 |
| Bronchitis | 142 |
| Pleurisy | 21 |
| Pneumonia | 735 |
| Hydrothorax | 47 |
| Asthma | 229 |
| Consumption | 2039 |
| Dis. of lungs, &c. | 201 |
| Total Dis. of Lungs, &c. | 3434 |

| Causes of Death. | Total Deaths. |
|---|---|
| **Class IV.** | |
| Pericarditis | 7 |
| Aneurism | 7 |
| Dis. of the heart, &c. | 219 |
| Total Dis. of Heart, &c. | 233 |
| **Class V.** | |
| Teething | 200 |
| Gastritis, enteritis | 192 |
| Peritonitis | 20 |
| Tabes mesenterica | 56 |
| Ascites | 4 |
| Ulceration | 18 |
| Hernia | 21 |
| Colic or ileus | 29 |
| Dis. of stomach, &c. | 55 |
| Hepatitis | 13 |
| Jaundice | 27 |
| Dis. of the liver, &c. | 109 |
| Total Dis. of Stom. &c. | 744 |
| **Class VI.** | |
| Nephritis | 4 |
| Diabetes | 3 |
| Stone | 5 |
| Stricture | 3 |
| Dis. of the kidneys, &c. | 58 |
| Tot. Dis. of Kidneys, &c. | 73 |
| **Class VII.** | |
| Childbed | 72 |
| Ovarian dropsy | 6 |
| Diseases of uterus, &c. | 42 |
| Tot. Dis. of Uterus, &c. | 120 |
| **Class VIII.** | |
| Rheumatism | 23 |
| Diseases of joints, &c. | 31 |
| Total Dis. of Joints, &c. | 54 |

| Causes of Death. | Total Deaths. |
|---|---|
| **Class IX.** | |
| Ulcer | 3 |
| Fistula | 2 |
| Diseases of skin, &c. | 7 |
| Total Dis. of Skin, &c. | 12 |
| **Class X.** | |
| Inflammation | 57 |
| Hemorrhage | 51 |
| Dropsy | 390 |
| Abscess | 35 |
| Mortification | 55 |
| Scrofula | 39 |
| Carcinoma | 93 |
| Tumour | 23 |
| Gout | 15 |
| Atrophy | 69 |
| Debility | 250 |
| Malformations | 11 |
| Sudden deaths | 166 |
| T. Dis. of Uncert. Seat. | 1254 |
| **Class XI.** | |
| Old age or nat. decay | 711 |
| **Class XII.** | |
| Intemperance | 12 |
| Privation | 4 |
| Violent deaths | 283 |
| Total by Violence, &c. | 299 |
| **Class XIII.** | |
| Causes not specified | 42 |
| T. Deaths from all causes<br>Males 5374, Females 5163 | 10537 |

### TEMPERATURE DURING THE QUARTER.

| | Weeks ending | | | | | | | | | | | | |
|---|---|---|---|---|---|---|---|---|---|---|---|---|---|
| | April | | | | May | | | | | June | | | |
| | 3d | 10th | 17th | 24th | 1st | 8th | 15th | 22d | 29th | 5th | 12th | 19th | 26th |
| Highest | 60 | 56 | 57 | 59 | 72 | 65 | 72 | 76 | 79 | 82 | 69 | 80 | 87 |
| Lowest | 38 | 38 | 37 | 40 | 44 | 50 | 46 | 48 | 54 | 52 | 45 | 47 | 51 |
| Daily mean | 49 | 48 | 46 | 48 | 59 | 58 | 58 | 59 | 65 | 66 | 54 | 61 | 64 |

Quarterly Maximum .... 87° Minimum .... 37° Daily Mean .... 55°

